# Post-interval potentials in temporal judgements

**DOI:** 10.1007/s00221-023-06568-y

**Published:** 2023-02-21

**Authors:** Ezgi Özoğlu, Roland Thomaschke

**Affiliations:** Cognition, Action, and Sustainability Unit, Department of Psychology, Albert-Ludwigs University of Freiburg, Freiburg, Germany

**Keywords:** Temporal bisection, Temporal generalization, LPC, Stimulus offset

## Abstract

Research suggests that post-stimulus positive deflections could be associated with timing. We compared offset-locked potentials N1, P2, N1P2, and late positive component (LPC) in temporal generalization and temporal bisection—with visual probe intervals. In both tasks, the LPC amplitude decreased with the duration of the current probe interval. A larger LPC was found after shorter intervals, whereas other ERP amplitudes did not change between tasks or across durations. We also found that the LPC for different responses indicates subjective time. We discussed our findings in relation to theories of human timing.

**Supplementary Information:**

The online version contains supplementary material available at 10.1007/s00221-023-06568-y.

## Introduction

The majority of our cognitive functions require precise processing of temporal intervals. For example, to control the flow of turn-taking in a conversation, it is essential to precisely time the inter-turn gap, because it entails important communicative information (e.g., Levinson 2016). Likewise, driving requires highly accurate timing, for example, when taking a highway exit at a high driving speed (Walker et al. 2019). One well-established model to describe human time processing is the pacemaker-accumulator model (PA) (Simen et al. 2013; Treisman 2013). This model assumes an internal clock comprising three stages: a clock stage, a memory stage, and a decision stage. At the start of any timed interval, a pacemaker emits pulses (the clock stage). These pulses are then accumulated and stored in working memory for further cognitive processing (the memory stage), for example, for later comparison with a previously learned interval (the decision stage).

Behavioural timing research has generated a large variety of interval timing tasks (Wearden 2016). Most of these tasks have discrete windows for presenting the to-be-timed interval and exerting the response, enabling researchers to obtain time-related electrophysiological markers (Grondin 2010), and interpreting cognitive timing models (e.g., PA models) in a neurobiological manner (Ng et al. 2011). Perceived time is usually measured from the onset time of the reference stimulus, whether this is a tone, a picture, or a letter. While it carries essential information regarding what happens during the perception phase, perceiving is not yet complete until the end of the stimulus. When stimuli do essentially evolve during the time, offset-locked EEG signals should be at least equally important for understanding the processing of these stimuli, because the full stimulus information is only present after the presentation. In that sense, one can expect the presented temporal information (objective time) and perception of this information (subjective time) to modulate potentials after stimulus offset. Previous studies suggested that post-stimulus positive deflections such as P2 and late positive component (LPC) could have the potential to explain timing behaviour (Bueno and Cravo 2021; Tarantino et al. 2010; Kruijne et al. 2021).

Several studies focussing on post-stimulus potentials in windows similar to LPC in timing tasks indicated that perceived time modulates this activity. Bueno and Cravo (2021) used a duration discrimination task (compared with a colour discrimination task) and analysed ERPs in two windows: an earlier (200–300 ms) parieto-occipital activity and a later (300–500 ms) fronto-central activity, resembling P2 and LPC, respectively. Parietal ERP followed a monotonic relation to proportional time; later, frontal activity showed an inverse relationship between mean amplitude and time. As S2 became shorter than S1, a larger frontal ERP was observed. Another post-stimulus deflection was found by Tarantino et al. (2010) in a duration discrimination task. This ERP, termed P150 by the authors, showed a linear increase by comparison interval at central and parietal sites and an inverse decrease at frontal and frontopolar sites. This positivity is interpreted as the processing of the elapsed time perceived by the participants. A similar pattern of relationship between LPC and interval was reported by Gontier et al. (2008; 2009) and Wiener and Thompson (2015). Likewise, Paul et al. (2003) found that frontal LPC increased by the interval. In an auditory reproduction task, Damsma et al. (2021) found a fronto-central P2 (between 140 and 300 ms) after stimulus offset) increasing by duration. However, note that reproduction tasks do not require categorical responses like discrimination tasks (they rather ask for an imitation of intervals by the start/stop responses).

Could such post-stimulus brain activities be more involved in temporal decision making than in reflecting the duration-based information? In addition to the changes as a function of absolute duration, LPC (or positive deflections in similar windows) modulations partly reflect the subjective time. For example, in the above-mentioned study by Bueno and Cravo (2021), they also found that LPC predicted the response (answering “shorter” or “longer”) in the duration task (and not in the colour task), suggesting that the LPC modulation is more than a surprisal effect due to interval ends. Kruijne et al. (2021) used a duration discrimination task in which they compared intervals with changing or repeating markers (markers that indicate the start and end of time intervals). Intervals were perceived to be longer when presented with changing markers. Along with the dilation in perceived time, higher P2 (200–250 ms post-stimulus) was observed after “longer” responses. They did not find this effect for P3 (350–400 ms). A difference in post-stimulus positivity (per response) was observed in contexts where the perceived time was not manipulated. Many factors contributing to the decision phase (such as interval length, difficulty level, or the critical value needed to make the decision) differentiate how intervals are processed even in neutral task conditions. In a bisection task, Lindbergh and Kieffaber (2013) found that responding “long” led to shorter RTs and lower centro-parietal positive activity between 200 and 700 ms after stimulus offset (compared to “short” responses). They argued that for longer intervals, memory and decision-related processes end before the interval offset. Similarly, Bannier et al. (2019) compared post-stimulus activity between bisection and generalization tasks. They found lower RTs for longer intervals in both tasks. They also analysed a centro-parietal activity (200–800 ms post-stimulus) in six successive 100 ms-long windows. Between 200 and 300 ms, a larger activity for longer intervals was observed, whereas the following windows until 800 ms showed the opposite pattern. Both studies indicate that longer and shorter intervals are processed differently, where shorter intervals require additional resources before making the temporal decision.

Providing a comparison between two of the commonly used timing tasks, bisection and generalization, Bannier et al. 2019 reported that the duration effect was more pronounced in the bisection task during the post-stimulus window of 400–500 ms. They found a main effect of the task in four of the six successive windows; generalization led to a larger positive signal than bisection. The authors found that this difference is in line with behavioural studies suggesting that generalization tasks are cognitively more demanding than bisections (Ogden et al. 2019). In the present study, we compared post-stimulus ERPs, N1, P2, N1P2, and LPC, in visual generalization and bisection tasks. We expected both tasks to reveal duration-based modulation in these ERPs. Based on findings suggesting late positive suggestion involvement in the decision-making stage of timing, we expected changes in perceived time to be reflected in LPC modulations.

## Materials and methods

### Participants

Thirty-nine students at the Albert Ludwig University of Freiburg with normal or corrected-to-normal vision participated in the study and received €24 each. Three participants from the bisection data and three participants from the generalization data were excluded from the sample, either due to technical problems during recording or excessive muscle artefacts and blinking. The final sample had 36 participants for the bisection task (10 male, 35 right-handed, mean age = 26.64) and 36 participants for the generalization task (10 male, 35 right-handed, mean age = 25.82). Before participation, each participant signed an informed consent form.

### Procedure

Each participant completed two timing tasks: a temporal generalization task and a temporal bisection task with simultaneous EEG recording. E-Prime (Psychology Software Tools, USA) software was used for stimulus presentation and data collection. The order of the two tasks was counterbalanced across the participants. Each task lasted approximately 1 h. There was an obligatory 10 min break between tasks.

### Temporal generalization

**Fig. 1:**
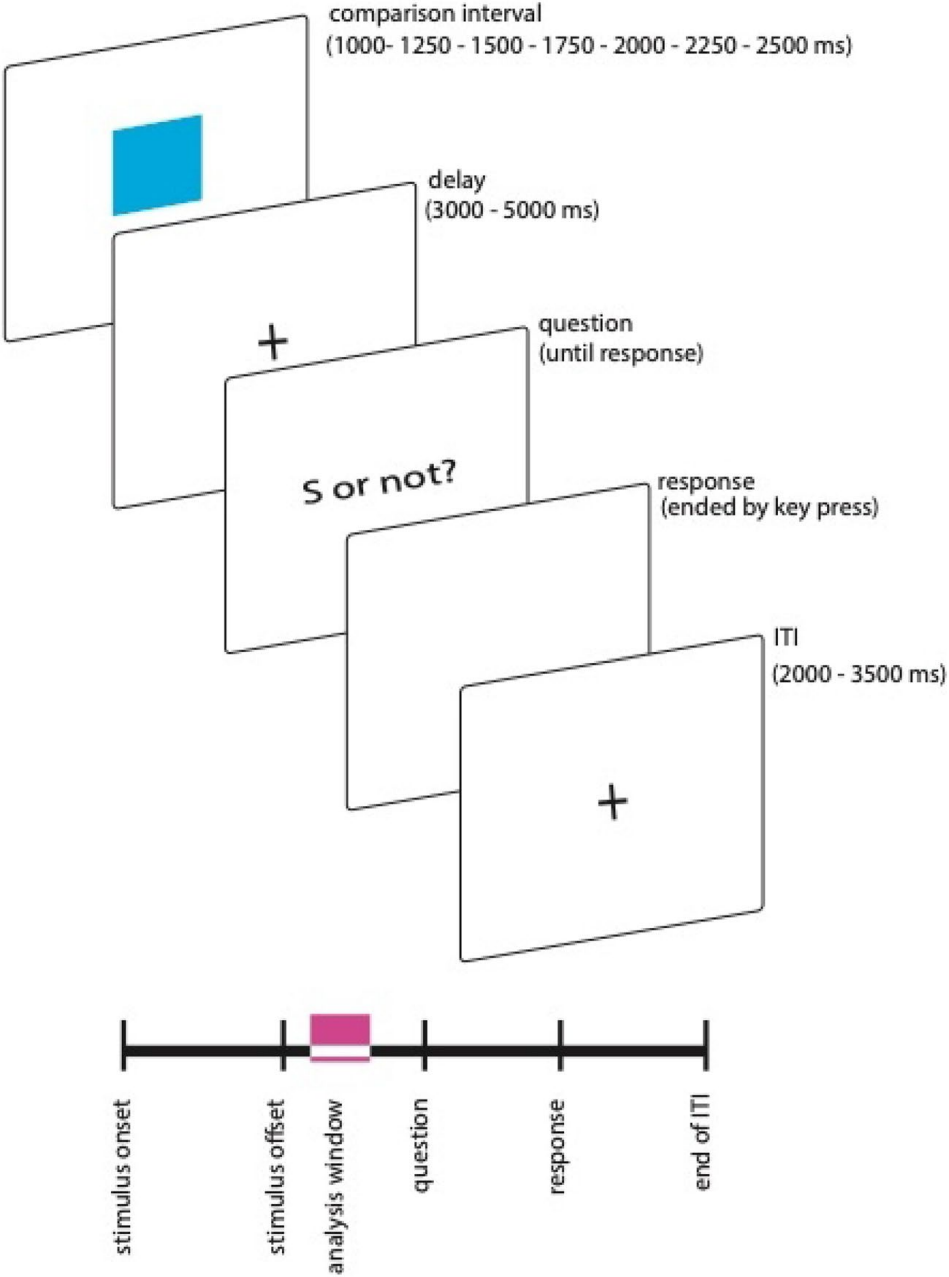
Trial sequence in generalization task. Pink shade on below timeline indicates EEG analysis window. See text for further details

The task divided into two blocks. Each block included a learning phase and a testing phase with the to-be-timed stimulus consisting of a 100 × 100 pixel blue square. In the learning phase, a reference duration (1750 ms) was presented 10 times. In the testing phase, in each trial, a probe interval was presented (randomly drawn from a set of intervals between 1000 and 2500 ms, i.e., 1000, 1250, 1500, 1750, 2000, 2250, and 2500 ms). The presentation was followed by a delay randomly selected between 3000 and 5000 ms to prevent motor response preparation. The task was to decide whether the probe interval was the same as the reference interval or different from the reference interval. Participants could respond only after the delay had elapsed, and when the question (‘Same or not?’) appeared on the screen. Participants responded via the keys X and M on a standard QWERTZ keyboard to indicate whether each probe was the same (left key X) or different (right key M). The response was followed by an intertrial interval (ITI), which was randomly chosen between 2000 and 3500 ms (see Fig. 1 for an illustration of a trial’s event sequence). In each block, participants completed 175 trials, 25 trials per probe interval, and 350 trials in total. There was a 5 min break between the blocks.

### Temporal bisection

**Fig. 2:**
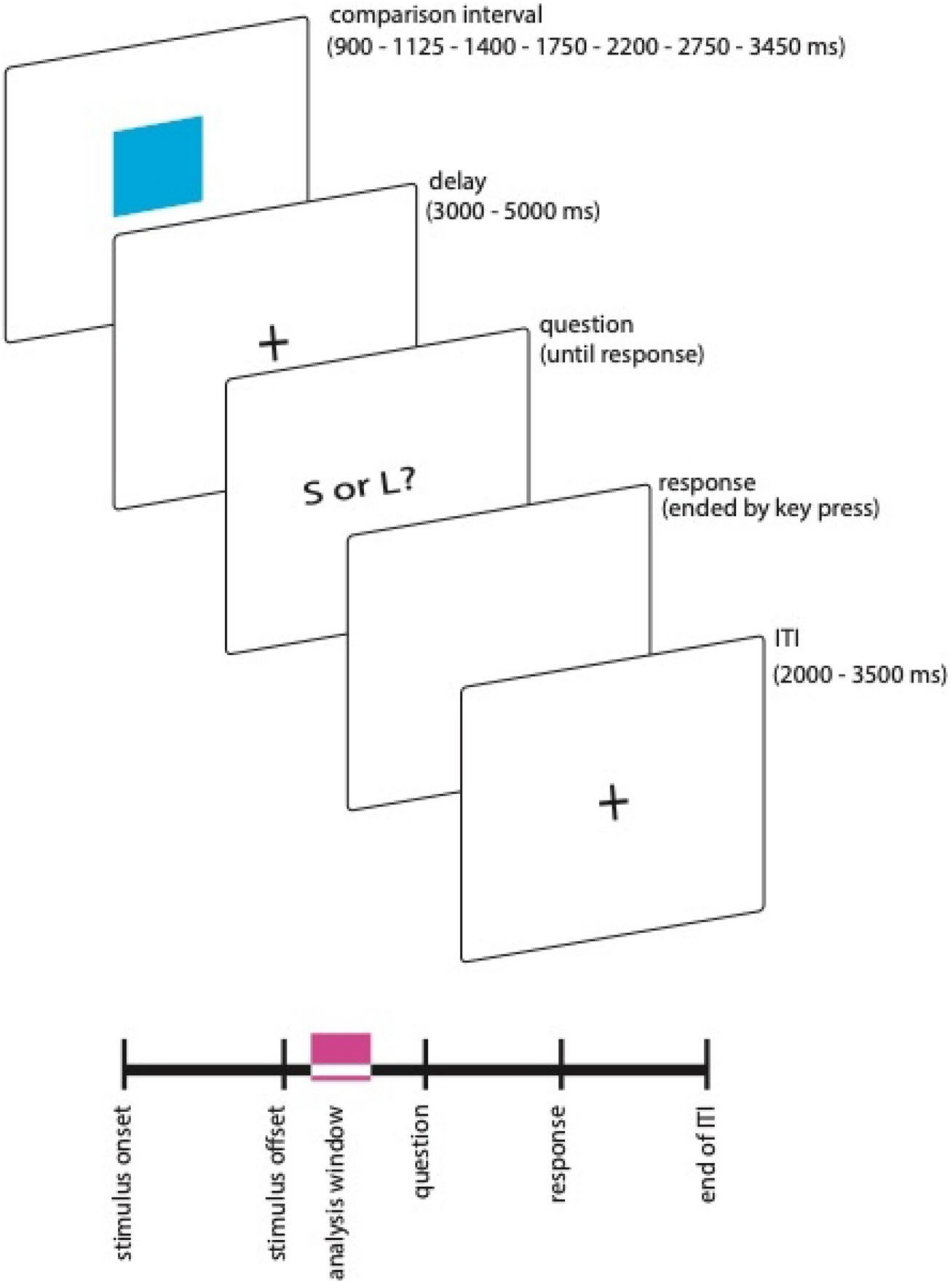
Trial sequence in bisection task. Pink shade on below timeline indicates EEG analysis window. See text for further details

The task consisted of two blocks. Each block included a learning phase and a testing phase. The time stimulus was presented with a 100 × 100 pixel blue square. In the learning phase, two standard durations (900 ms and 3450 ms) were randomly presented five times each. After each presentation, feedback was given on the duration (‘That was the short/long duration.’). In the testing phase, a comparison interval was presented in each trial. Comparison intervals were logarithmically spaced between 900 and 3450 ms (i.e., 900, 1125, 1400, 1750, 2200, 2750, and 3450 ms). Each interval appeared 25 times in each block. The order of the presented intervals was randomised. The comparison interval presentation was followed by a delay before the participants could respond. This delay was included to prevent motor response preparation during the interval (Damen and Brunia 1987). The delay was randomly selected between 3000 and 5000 ms. Participants could respond only after the end of the delay when the question (‘Closer to short or long?’) appeared on the screen. Participants responded via the keys X and M on a standard QWERTZ keyboard to indicate whether the probe was closer to the short (left key X) or long (right key M) standard, respectively. The ITI was randomly determined between 2000 and 3500 ms (see Fig. 2 for an illustration of a trial’s event sequence). In each block, participants completed 175 trials, 25 trials for each probe interval, and 350 trials in total. There was a 5-min break between the blocks.

### EEG data acquisition

The electroencephalogram (EEG) data were recorded from 32 electrode locations (FP1, FP2, FPZ F3, F4, F7, F8, FT9, FT10, FZ, FC1, FC2, FC5, FC6, T7, T8, TP9, TP10, C3, C4, CP1, CP2, CP5, CP6, P3, P4, P7, P8, PZ, O1, O2, OZ) with Ag/AgCl electrodes using BrainVision Recorder software (Brainproducts, Germany). The electrodes were attached to the cap with a standard 10–20 layout. Ground and reference electrodes were used in electrode sites FPZ and CZ, respectively. To record eye activities, vertical and horizontal electrooculogram (EOG) electrodes were located above and below the right eye and at the outer canthi of both eyes, respectively. Data were sampled at 500 Hz. Impedances were kept below 10 kΩ.

### Data analysis

EEG data were preprocessed using a BrainVision Analyser (Brainproducts). After down-sampling to 250 Hz, data were offline re-referenced to mastoid electrodes, and 40 Hz low-pass and 0.1 Hz high-pass filters and 50 Hz notch filters were applied. Ocular artefacts were removed via independent component analysis (ICA). A trial was discarded when the changing voltage exceeded 75 µV in any channel. On average, 2.8% (2.7%) of trials were discarded in bisection (generalization) data.

We focussed on offset-locked potentials and defined N1 as the mean voltage between 100 and 200 ms, P2 as the mean voltage between 200 and 300 ms, peak-to-peak N1P2 as the summation of absolute values of N1 and P2, and LPC as the mean voltage between 300 and 500 ms. Time stimuli lead to a negative ramping activity during the interval presentation, which can contaminate the offset potentials. To prevent this potential effect, we followed Kononowicz and van Rijn’s (2014) method and applied a 1–20 Hz filter. All trials were baseline corrected to the mean voltage of 50 ms preceding and following stimulus offset.

## Results

Both N1, P2, peak-to-peak N1P2, and LPC (averaged at the participant level) were analysed using linear mixed models (LMM), with duration as the predictor. A random effect per participant was added, and this improved the models (confirmed by LRT for random effects). For multiple comparisons Holm correction was applied when needed. For generalization data, duration did not predict P2 amplitude (*β* = − 0.02, SE = 0.07, *t* = − 0.32, *p* = 0.74), N1 amplitude (*β* = 0.03, SE = 0.05, *t* = 0.59, *p* = 0.55), or N1P2 amplitudes (*β* = − 0.01, SE = 0.09, *t* = − 0.10, *p* = 0.91). Yet, LPC decreased by duration well (*β* = − 0.32, SE = 0.05, *t* = − 6.25, *p* < 0.001). Pairwise comparisons confirmed differences between pairs 1000–2000, 1000–2250, 1000–2500, 1250–2250, 1250–2500, 1500–2250, 1500–2500, 1750–2250, 1750–2500, and 2000–2500 ms (all remaining *p* values were between 0.10 and 1.00). For bisection data, the results showed no effect of duration on P2 amplitudes (*β* = − 0.10, SE = 0.04, *t* = − 2.16, *p* = 0.51), N1 amplitudes (*β* = − 0.07, SE = 0.04, *t* = − 1.62, *p* = 0.10), or N1P2 amplitudes (*β* = − 0.19, SE = 0.10, *t* = − 1.86, *p* = 0.06). However, LPC decreased by duration (*β* = − 0.47, SE = 0.04, *t* = − 10.87, *p* =  < 0.001). Pairwise comparisons confirmed differences between pairs 1150–1750, 1150–2200, 1150–2750, 1150–3450, 1400–2200, 1400–2750, 1400–3450, 1750–2750, and 1750–3450 ms (all remaining *p* values were between 0.13 and 1.00).

**Fig. 3:**
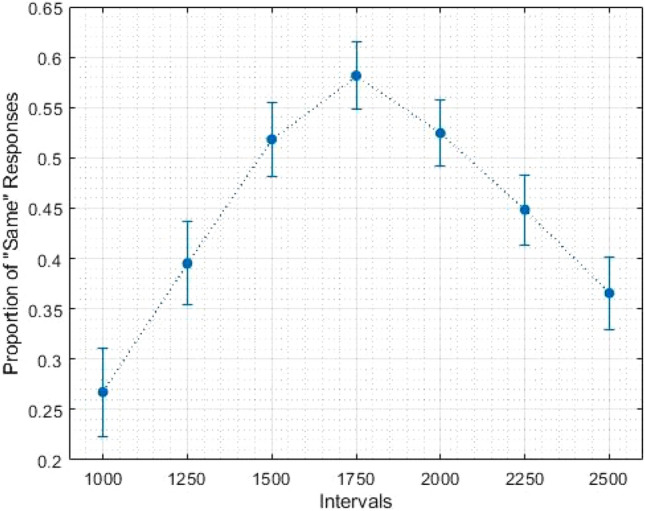
Average proportion of “same” responses in generalization task. Error bars represent standard error

**Fig. 4:**
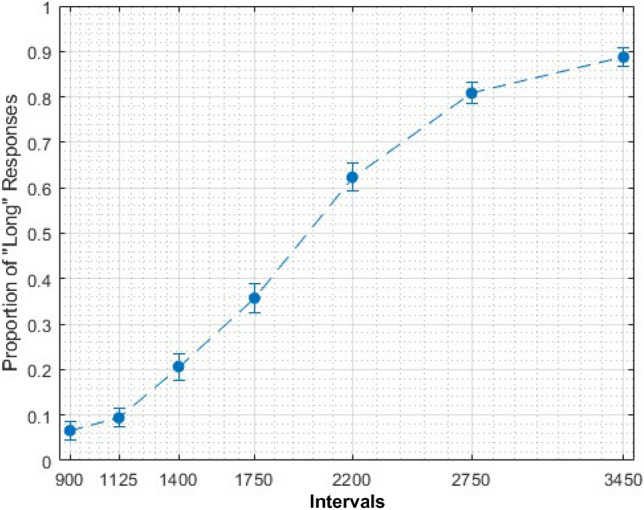
Average proportion of “long” responses in bisection task. Error bars represent standard error

We wanted to further investigate whether the common decrease in LPC data is linked to temporal processing. For each participant, we fitted a regression line on the LPC data (by average amplitude per duration—from shortest to longest interval). For the generalization task, we used this data to correlate with behavioural parameters. To do so, we fitted each individual’s proportion of the “same” responses (Fig. 3) to a log-normal (amplitude) function, using the Peakfit Program (Peakfit 4.12 for Windows). From each fitting, we obtained the full width at the half maximum (FWHM) and centre (the position of the peak) as an index of temporal sensitivity and temporal accuracy, respectively (for a similar approach see Cocenas-Silva et al. 2014). We have correlated these values with the coefficient from the fit on LPC data, and found that lower FWHM was associated with greater coefficients [*r*(35) = 0.36, *p* < 0.05]. We have no association between centre and coefficient values. For the bisection task, we fitted each individual’s proportion of the “long” responses (Fig. 4) to a cumulative Gaussian function and calculated the Weber fraction (WF) and the point of subjective equality (PSE) as indexes of temporal sensitivity and temporal accuracy, respectively (see Kopec and Brody 2010). We correlated the temporal correlated Wf and PSE with the regression coefficient obtained from the LPC data. We found that lower WFs were associated with greater regression coefficients [*r*(35) = − 0.47, *p* < 0.05]; and found no association between PSE and coefficients [*r*(35) = − 0.11, *p* = 0.50].

**Fig. 5:**
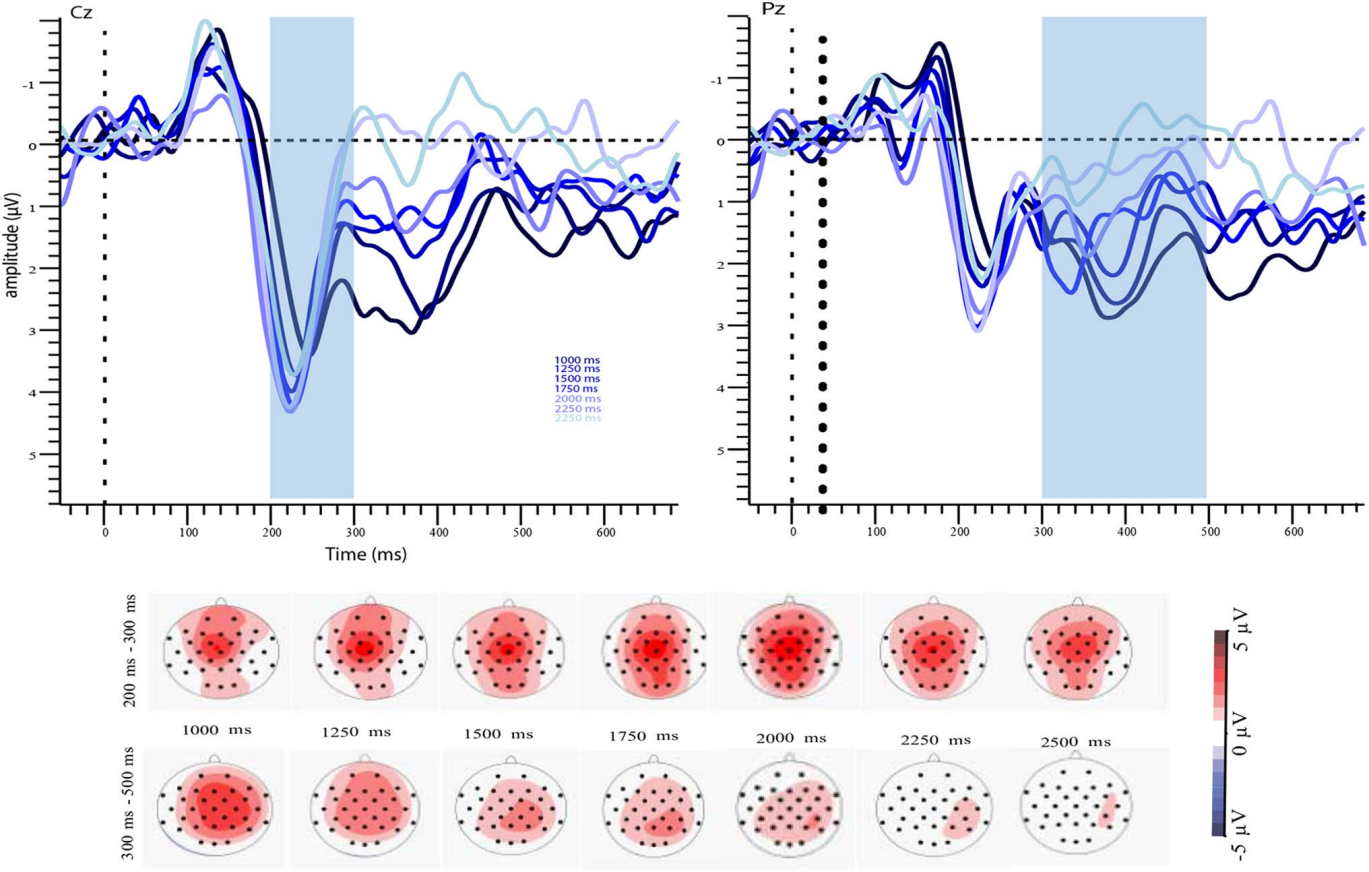
Grand-averaged ERPs per comparison interval in generalization task. Blue shade indicates the analysis window. Each line shows average waveform of comparison interval. Topographic plots below show average voltage during P2 (upper) and LPC (lower) measures

**Fig. 6:**
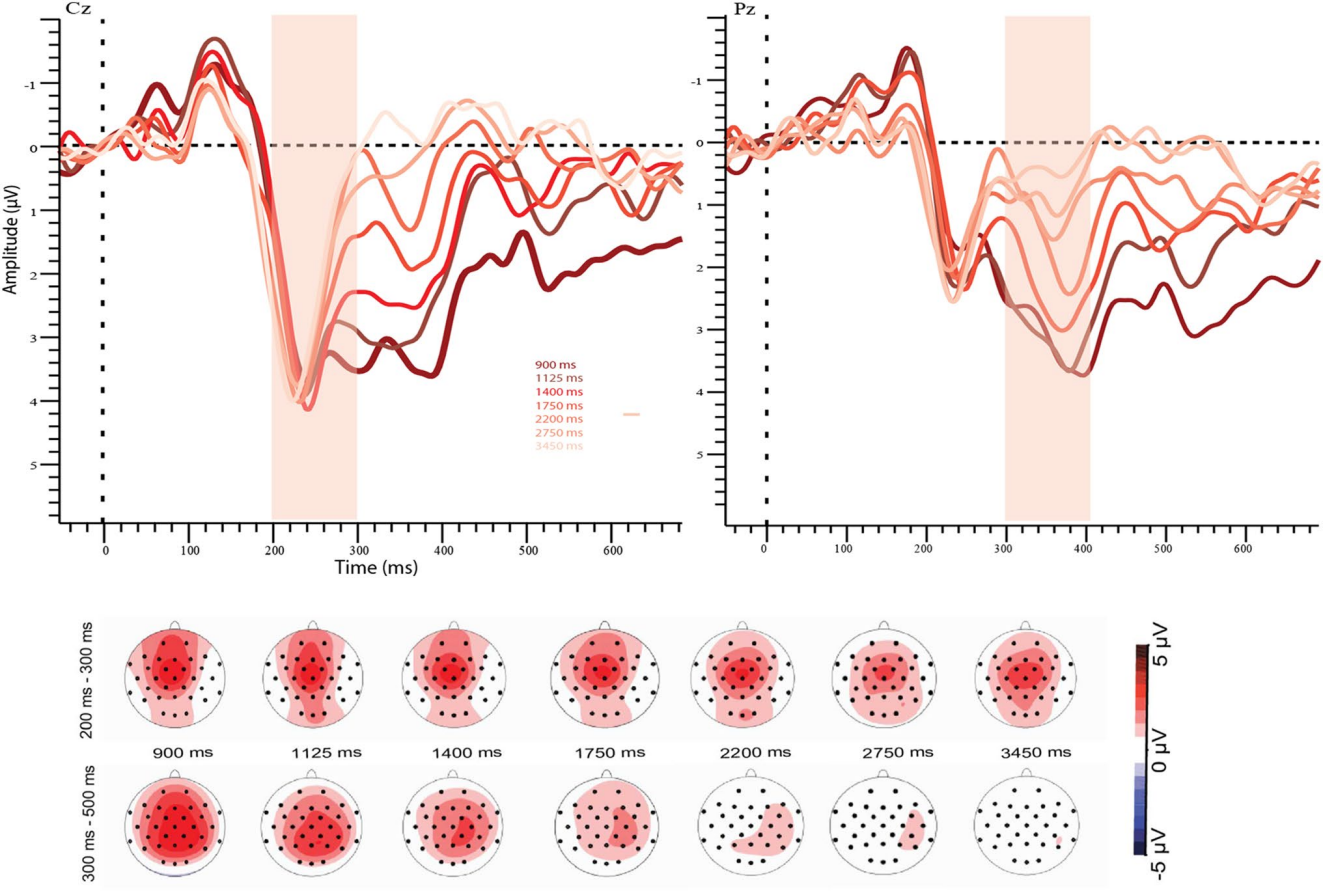
Grand-averaged ERPs per comparison interval in bisection task. Pink shade indicates the analysis window. Each line shows average waveform of comparison interval. Topographic plots below show average voltage during P2 (upper) and LPC (lower) measures

**Fig. 7:**
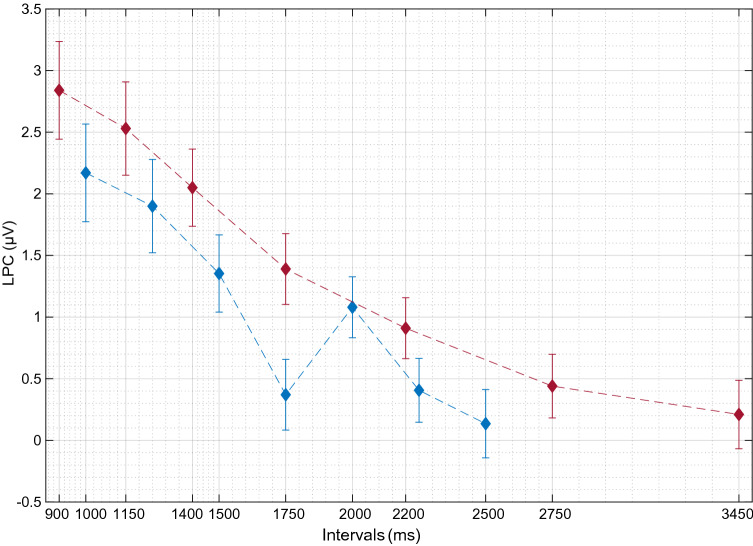
Participant-level LPC averages. Red line shows bisection data, blue line shows generalization data. Error bars represent standard error

**Fig. 8:**
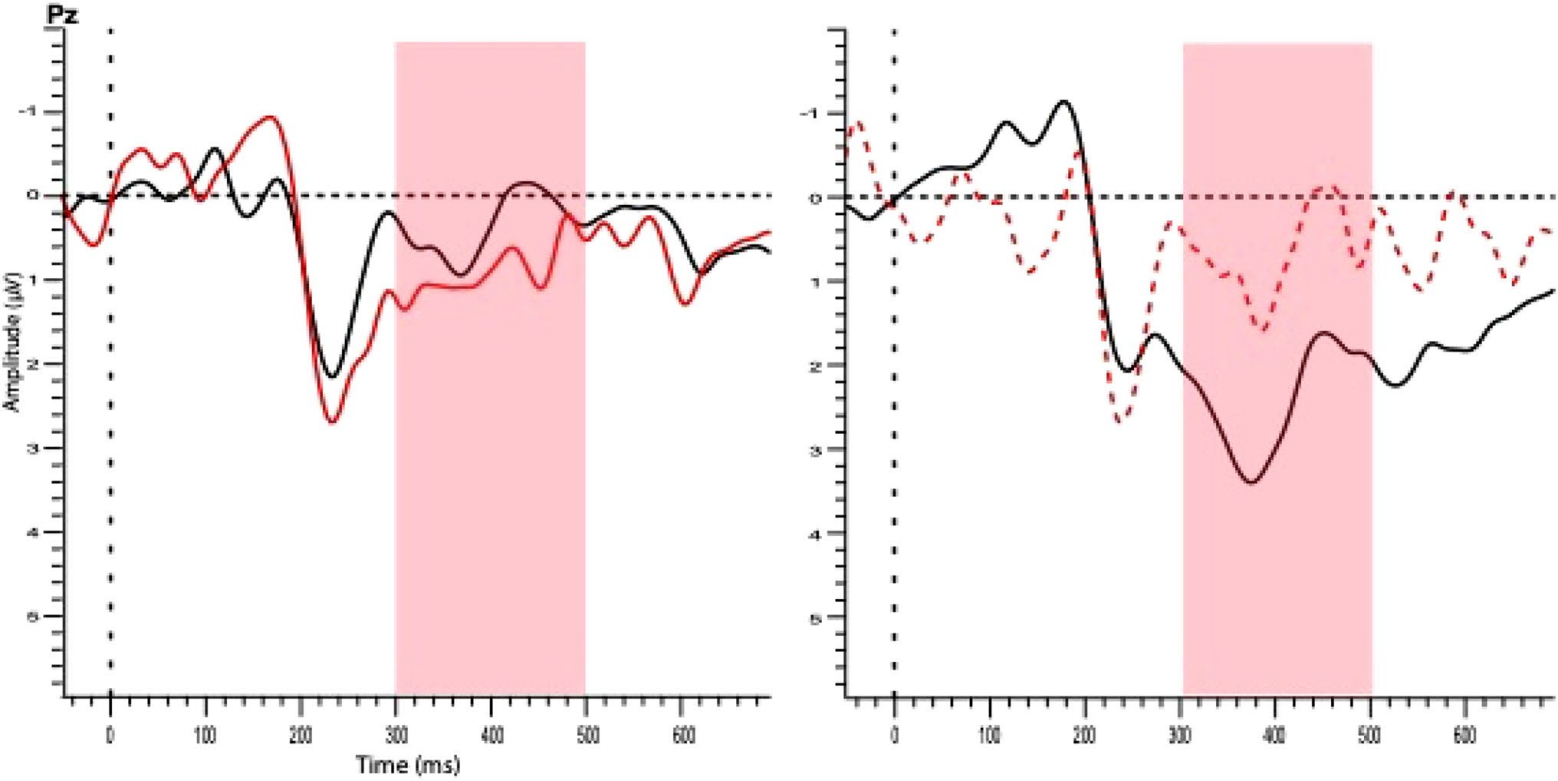
Grand-averaged waveforms for response types in bisection task. Waveform on the left compares correct (black line) vs overestimated (red line) trials using shorter comparison intervals (900, 1150, 1400 and 1750 ms). Waveform on the right compares correct (black line) vs underestimated (red dashed line) trials using longer comparison intervals (2200, 2750, 3450 ms). Pink shade shows LPC analysis window

**Fig. 9:**
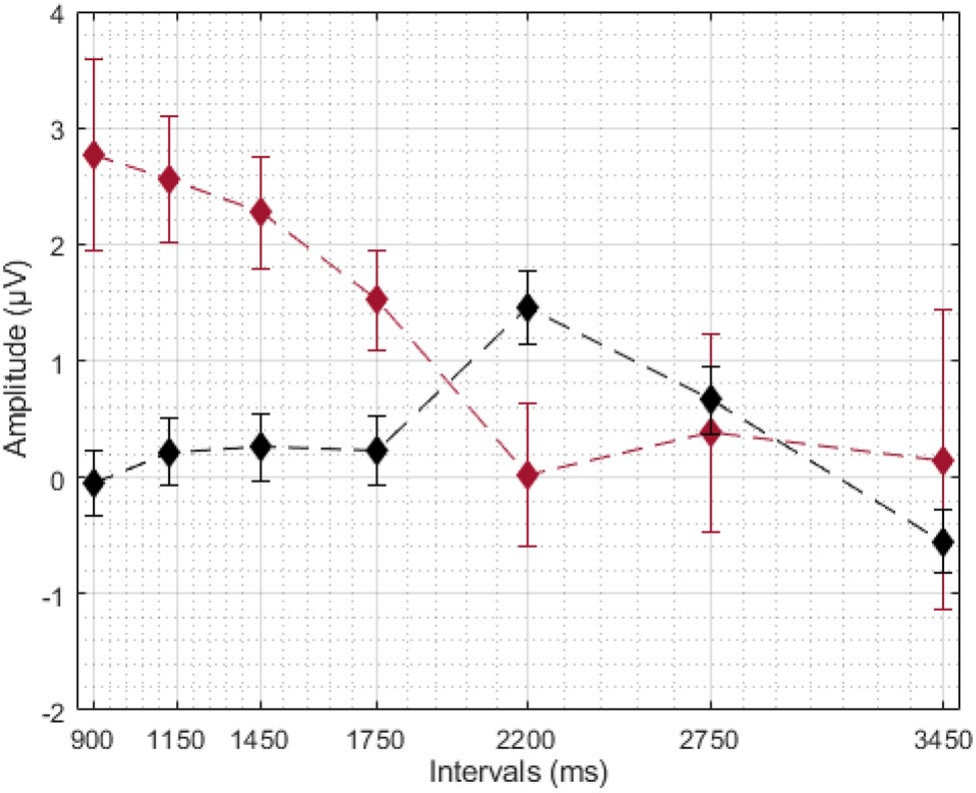
Average LPC amplitudes per interval for correct (black) and incorrect (blue) responses in bisection data. Error bars represent standard error

**Fig. 10:**
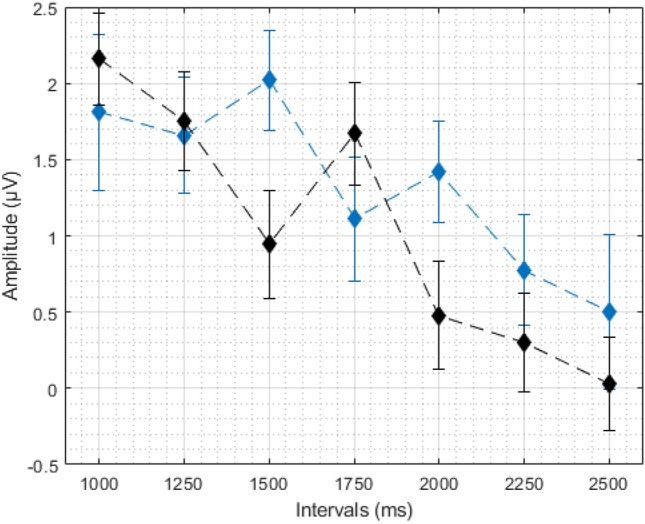
Average LPC amplitudes per interval for correct (black) and incorrect (red) responses in generalization. Error bars represent standard error

The main effect of duration on LPC showed that as the absolute duration of the comparison interval increased, the amplitude of the LPC signal decreased (Fig. 5 and Fig. 6 for generalization and bisection, respectively; and Fig. 7 for both tasks). We wanted to further investigate whether the LPC is changing when the absolute duration is the same, but it was subjectively classified as longer (or shorter) than it is. Grand-averaged plots indicated a difference between overestimated (underestimated) and correct trials. Due to the natural imbalance in the number of correct and incorrect responses, we ran an LMM with single-trial values of LPC, with accuracy as the predictor. Since we know that duration affects LPC, we added it as a fixed-effect factor. We added a random intercept per participant. As longer intervals lead to lower LPC, we expected “subjective longs” (overestimated trials) to result in lower LPC, and the opposite pattern between underestimated and correct trials. In bisection data, there was no main effect of duration (*β* = 0.04, SE = 0.09, *t* = 0.43, *p* = 0.68), a significant effect of accuracy (*β* = 2.93, *SE* = 0.45, *t* = 6.48, *p* < 0.001), and an interaction effect (*β* = − 0.56, SE = 0.10, *t* = − 5.52, *p* < 0.001; Fig. 8). Simple effect analysis showed that LPC in the overestimated trials was lower than in the correct trials for all four intervals: 900, 1150, 1400, and 1750 ms (all *p* < 0.05); and LPC in the underestimated trials was higher than in the correct trials for 2200 ms (*p* < 0.05), but not for 2750 and 3450 ms (*p* = 0.27 and *p* = 0.98, respectively; Fig. 9). In the generalization task, incorrect responses do not necessarily indicate under- or overestimation of intervals. Therefore, we could not run the same analysis. However, you can refer to the supplementary materials for a similar analysis of generalization data (Fig. 10).

To compare the LPC and P2 between tasks, we selected the common interval in both tasks (1750 ms) and ran an LMM on single-trial LPC and P2 values. For both models, we added task and accuracy as predictors and gave a random intercept per participant. The LPC results showed a main effect of task (*β* = 0.94, SE = 0.38, *t* = 2.43, *p* < 0.05); the generalization task led to a higher LPC than the bisection task. Accuracy had a significant effect with lower amplitudes for “long” responses (*β* = 2.94, SE = 0.77, *t* = 3.81, *p* < 0.001). There was an interaction between task and accuracy (*β* = − 1.34, SE = 0.49, *t* = − 2.72, *p* < 0.01), showing that “long” responses did lead to lower amplitudes in the bisection task (*p* < 0.05), but not in the generalization task (*p* = 0.45). The P2 results showed no effect of task (*β* = 0.19, SE = 0.48, *t* = 0.40, *p* = 0.31) or accuracy (*β* = 0.36, SE = 0.95, *t* = 0.38, *p* = 0.61) and no interaction effect between task and accuracy (*β* = 0.23, SE = 0.60, *t* = 0.39, *p* = 0.63).

## Discussion

In this study, we investigated post-stimulus ERPs, N1, P2, N1P2, and LPC, that have been previously associated with timing. We used two tasks: temporal bisection and generalization, sharing a common comparison interval. In line with previous findings, our results showed that post-stimulus LPC relates to the magnitude of the time stimulus (absolute time) and to the changes in perceived time.

Comparing ERPs over intervals, we found that the duration of the comparison interval had an inverse relationship with the offset-locked LPC amplitude, but not P2. Further analysis of LPC per response accuracy showed that this modulation is associated with temporal judgement, rather than passively reflecting the expectancy-based reaction to the end of the stimulus.

In both tasks, LPC—but not P2—showed a gradual decrease in duration. After shorter intervals, there was a larger LPC, which decreased as the comparison interval became longer. It has been suggested that after long intervals, decision-making has already taken place at the offset, whereas for shorter intervals, the process is not yet complete (Bannier et al. 2019; Lindbergh and Kieffaber 2013) which is reflected in larger LPC amplitudes. This explanation links LPC (or positive deflections in the same temporal window) to the temporal decision-making process, and our findings are in line with this view. We have analysed all the trials in this analysis, but note that correct and incorrect judgements depicting differing patterns across durations. Additionally, we found that a steeper decrease in the LPC trend indicated higher temporal sensitivity in both tasks.

Our results showed that LPC amplitude differed between temporal responses in a similar way to duration-based modulation. When the same interval was incorrectly classified as “long”, it showed lower amplitudes as compared to correct classifications. Similarly, subjective “short” classifications led to higher LPC amplitudes. However, note that this effect was found by grouping all durations. When compared in a pairwise fashion, this effect holds for 2200 ms and not for 2750 ms and 3450 ms.

In a recent study, Kruijne et al. (2021) showed a similar post-stimulus potential (called P3) that decreased by duration but was not related to behaviour. However, they found a fronto-central P2 that linked to time behaviour, with larger amplitudes after “long” responses. In another recent study by Bueno and Cravo (2021), a pario-central P200 was not linked to time or behaviour, and a fronto-central LPC decreased by duration and was associated with behaviour. The pattern of LPC in our study resembles these findings regarding its relation to duration and supports the proposal that LPC is linked to temporal decision-making. However, it should be noted that the LPC (and P2) measured in these different studies correspond to varying tasks, topographies, temporal windows, and task manipulations.

One other study that compared temporal bisection and generalization is Bannier et al. (2019). They also found a centro-parietal wave between 200 and 700 ms post-stimulus, which overlaps with our analysis window for P2 and LPC. When we compared P2 and LPC (for 1750 ms) between tasks, we found overall higher LPC amplitudes in generalization than bisection. Similar to our findings, they found larger amplitudes between 200 and 300, 300 and 400, 500 and 600, and 700 and 800 ms. They interpreted this finding (along with shorter RTs in bisection) as a generalization requiring more cognitive resources to make the decision. Regarding the effect of duration, they found larger amplitudes after shorter intervals in the 300–400, 500–600, and 600–700 ms windows, resembling our LPC modulation. They found the opposite pattern in 200–300 ms, which corresponds to our P2 window. Their interaction effect in 400–500 ms showed that this finding was pronounced in the bisection task. While we also observed more prominent differences in the bisection tasks, it should be noted that this difference could be driven by the cognitive demands of the tasks or by the different stages of decision-making taking place after the offset. The critical information needed to make the decision in the bisection task is an open question, and future studies are needed to clarify whether differences in neural correlates are due to how time information is processed in each task.

In conclusion, our findings suggest that post-stimulus LPC indicates objective time and at least partly subjective temporal judgements both in bisection and generalization tasks. Our results also suggest that differences in task demands in bisection and generalization can modulate LPC, though future studies with controlled comparisons are needed to understand the task properties’ relation to post-stimulus activity across timing tasks.

## Supplementary Information

Below is the link to the electronic supplementary material.

Supplementary file1 (DOCX 12 KB)
