# Supplementary material for: Post-interval potentials in temporal judgements
Source: Exp Brain Res. 2023 Feb 21;241(3):917–26. doi: 10.1007/s00221-023-06568-y (PMC9985573; doi:10.1007/s00221-023-06568-y)
Supplement: Supplementary file 1 — Supplementary file1 (DOCX 12 KB) [file 221_2023_6568_MOESM1_ESM.docx]

Supplementary Materials

In generalization task, results showed main effects of duration (*β* = -0.15, *SE* = 0.05, *t* = -2.74, *p* < .05), no effect of accuracy (*β* = 0.57, *SE* = 0.31, *t* = 1.84, *p* = .13), and an interaction between duration and accuracy (*β* = -0.26, *SE* = 0.06, *t* = -3.80, *p* < .05). Simple effect analysis showed that LPC in incorrect trials was higher than it was incorrect trials for comparison intervals 2000 and 2500 ms (for bot pairs h *p*<.05). In addition, LPC after 1500 ms was higher in incorrect trials (*p*<.05).
